# On the Kaolinite Floc Size at the Steady State of Flocculation in a Turbulent Flow

**DOI:** 10.1371/journal.pone.0148895

**Published:** 2016-02-22

**Authors:** Zhongfan Zhu, Hongrui Wang, Jingshan Yu, Jie Dou

**Affiliations:** 1 College of Water Sciences, Beijing Normal University, Beijing, China; 2 Department of Natural Environmental Studies, The University of Tokyo, Kashiwa, Japan; CNRS, FRANCE

## Abstract

The flocculation of cohesive fine-grained sediment plays an important role in the transport characteristics of pollutants and nutrients absorbed on the surface of sediment in estuarine and coastal waters through the complex processes of sediment transport, deposition, resuspension and consolidation. Many laboratory experiments have been carried out to investigate the influence of different flow shear conditions on the floc size at the steady state of flocculation in the shear flow. Most of these experiments reported that the floc size decreases with increasing shear stresses and used a power law to express this dependence. In this study, we performed a Couette-flow experiment to measure the size of the kaolinite floc through sampling observation and an image analysis system at the steady state of flocculation under six flow shear conditions. The results show that the negative correlation of the floc size on the flow shear occurs only at high shear conditions, whereas at low shear conditions, the floc size increases with increasing turbulent shear stresses regardless of electrolyte conditions. Increasing electrolyte conditions and the initial particle concentration could lead to a larger steady-state floc size.

## Introduction

In contrast to non-cohesive sediments such as sand, gravel and cobble, cohesive sediments possess complex electro-chemical and biological-chemical characteristics. These sediments carry charges on their surfaces and can absorb some pollutants (such as heavy metals) and nutrients owing to electro-chemical attraction of clay particles and/or organic matter in the sediment[1–2]. Under a shear flow environment in rivers, estuaries and coastal zones, cohesive fine-grained sediment can aggregate into different-sized flocs through the collision and the bonding between primary particles. However, the shear flow may disrupt those fragile and loose flocs, and as a result, some flocs may disaggregate into smaller flocs/primary particles [3–4]. This dynamic process has been termed to be flocculation induced by the flow shear (or orthokinetic flocculation) [3,5]. The flocculation process depends on the physico-chemical properties of the sediment and the water as well as the flow shear. Relative to primary particles, the flocs formed in the water-sediment suspension have larger sizes, low excess densities and high settling velocities. Because the adsorption process and the transport rate of some pollutants and nutrients are functions of these parameters, it is of vital importance to investigate the floc properties in a turbulent flow.

The flocculation process of cohesive sediment and other particles (such as polystyrene/latex particles) in a fluid has been studied by physicists, chemical engineers and sanitary engineers [e.g. 6–11]. The shear-induced flocculation process was studied theoretically using some heuristic analytical models [2,12–14]and a series of numerical models [15–19]. All of these models were based on a classical basic equation originally proposed by Smoluchowski (1917), which describes the rate of change of the number concentration for particles of a certain size as a result of the gain due to flocculation of particles of smaller sizes and the loss due to its flocculation with particles of other sizes under a shear flow [20]. Breakage of large flocs into smaller units also affects the concentration of a size group. In addition to these theoretical studies, some experiments reported two typical kinds of behaviour showing the temporal variation of floc size during the shear-induced flocculation process. In the first behaviour [1,7,21–30], the median floc size grows rapidly with time at the beginning of the flocculation experiment. This is a result of increased production of large flocs due to collisions and adhesions between primary particles/smaller flocs induced by the flow shear. The rapidity with which the median floc size increases with time then decreases; that is, the floc size experiences a slower increase with increasing flocculation time. This is because those large flocs possess fragile and loose structures and thus are susceptible to the breakage induced by the flow shear. Finally, the median floc size reaches an equilibrium. We referred to this trend of the temporal variation of the floc size as trend A. In another behaviour [16,22,30–34], the median size first undergoes a rapid increase to a peak value with time and then decreases again until a steady state is approached. The reason for the appearance of the maximum value in the floc size *vs*. time curve was regarded as a possible result of breakages and/or restructurings(or breakages and re-aggregations) of the flocs in a recent study of Bubakova et al. (2013) after Selomulya et al.(2001) [30,32]. We refer to this trend of the temporal variation of the floc size as trend B in this study. The variation of the median floc size at the steady state of flocculation with respect to various flow shear conditions was also a focus of some experimental studies. These experiments reported that the median floc size decreases as the shear stress increases [6–7,21,25,30,33,35–38]. Furthermore, a power function was used to describe this dependence as follows:

$$\begin{array}{ccc} mean & floc & size=c(\begin{array}{ccc} flow & shear & parameter{)}^{-\gamma } \end{array} \end{array}$$ (1)

where *c* is the floc strength that strongly depends on the method used to measure the floc size, and *γ* is the stable floc size exponent. For these experimental results, we must pay great attention to the following two questions in the context of the shear-induced sediment flocculation study:

(1) Large shear conditions were used in the reported experiments (the shear rate or shear velocity gradient,*G*, a parameter commonly used for characterizing the shear flow, was found to be larger than 25–30*s*^*-1*^);in contrast, insufficient attention has been paid to particle flocculation in low shear flows.
(2) The impacts of initial particle concentration and electrolyte condition on flocculation characteristics of cohesive sediment were still unclear.

This study attempts to investigate how floc size varies with respect to flow shear stresses over a relatively wide range at the steady state of flocculation, with a focus on low shear stresses, by measuring the kaolinite floc size during a Couette-flow experiment through sampling observation and an image analysis system. The effect of initial sediment concentration on the steady-state floc size and electrolyte condition is also studied. Section 2 briefly introduces the experiment. Section 3 discusses the effects of six flow shear conditions, the electrolyte condition, and the initial particle concentration on the steady-state floc size. Section 4 presents two conclusions.

## Experimental Introduction

Similar to some reported experiments [7,30,39], we used a Couette-flow system because it can yield a more isotropic turbulent shear flow compared with other devices, such as an impeller mixer [25,35] and an oscillating grid [40–41]. This system consisted of two concentric cylinders: the inner cylinder had a radius of 150*mm*, the outer one had a radius of 236*mm*, and the heights of both cylinders were 682*mm*. The outer cylinder was stationary, whereas the inner one was rotated at different angular velocities, thereby generating different flow shear environments where flocculation occurred. A speed-adjusting motor, coupled with different speed reduction boxes, was responsible for adjusting the angular velocities of the inner cylinder, *ω*, which ranged from 1.8 revolutions per minute (*rpm*) to 180*rpm*.

In this experiment, we used an acoustic doppler velocity-meter (ADV)(produced by SonTek Corporation, USA) to measure the flow field when the angular velocity of the inner cylinder was continually increased. The ADV was equipped with three probes to measure radial, tangential and vertical components of the velocity in the cylindrical coordinate system in the Couette-flow. Because of the limitations of ADV probes and the gap between the outer and inner cylinders, we can measure the velocities of only some points along the vertical direction in the centre of the gap of the cylinders (the number of locations at which the measurements were taken was 8–9, and they were uniformly distributed along the vertical direction). For Newtonian fluid in the cylinders in which the outer cylinder is fixed and the inner one rotates, it was reported that as the angular velocity of the inner cylinder increases from the stationary state, the fluid experiences a series of transitions of flow regimes: laminar Couette flow, laminar Taylor vortex flow, wavy vortex flow, turbulent vortex flow, and turbulent flow [42]. In this study, the spectral analysis of velocity data measured by ADV was simply adopted to estimate the critical angular velocity of the inner cylinder at which the flow in the Couette-flow system became turbulent [43],*ω*_*c*_. The value was *ω*_*c*_ = 27*rpm*, and the determination of this value was based on the high-frequency end of a spectrum (a detailed description can be found in Zhu (2009)) [44]. Considering that the angular velocities of the inner cylinder provided by the speed-adjusting motor coupled with speed reduction boxes can be adjusted only in the sequential order—18 *rpm*, 24 *rpm*, 27 *rpm*, 42 *rpm*, 60 *rpm*, 90 *rpm*, 120 *rpm*, 150 *rpm* and 180 *rpm*—we simply set them to be 42 *rpm*, 60 *rpm*, 90 *rpm*, 120 *rpm*, 150 *rpm* and 180*rpm* in this experiment (the absence of the values 18 *rpm*, 24 *rpm* and 27*rpm* was because only the influence of various turbulent shear conditions on the flocculation is studied in this work). These values were simply chosen so that they cover a range from low to high turbulent shear stresses, as will be shown in the estimations of characteristic turbulent fluctuating velocity in the following and *G* in Section 3.2, thus satisfying the requirement of this experiment.

The turbulent fluctuating velocity seemed to be suitable to characterize the shear flow, and the procedure for determining it was as follows. (1)Based on the measured velocity data at a measured point *I*, the radial, tangential and vertical turbulent fluctuating velocity components (*u*_*I1*_,*u*_*I2*_,*u*_*I3*_) can be calculated. (2)The turbulent fluctuating velocity at this measured point,*u*_*I*_, can be estimated using the following expression [43]:

$$u_{I}=\sqrt{\left(u_{I1}^{2}+u_{I2}^{2}+u_{I3}^{2}\right)/3}$$ (2)

(3) The characteristic turbulent fluctuating velocity corresponding to a flow shear environment,$\bar{u}$, can be determined by averaging the turbulent fluctuating velocity values for all measured points as $\bar{u}=\sqrt{\sum_{I=1}^{N}u_{I}^{2}/N}$(here, *N* = 8 − 9 is the number of locations at which the measurements were taken, as already introduced). The results showed that the vertical distribution of the turbulent fluctuating velocity was approximately uniform over the vertical range of the Couette-flow system. All data (on velocities and calculated turbulent fluctuating velocities) corresponding to different angular velocities of the inner cylinder can be found in Zhu(2009)(S1 Supporting Information)[44]. The aforementioned procedures provided an approximate estimation of the turbulent fluctuating velocity, and we used them only as a basis for comparing the impacts of various turbulent shear conditions on flocculation characteristics. Table 1 presents the calculated characteristic turbulent fluctuating velocity values corresponding to all angular velocities of the inner cylinder used in the present study. As expected, the characteristic turbulent fluctuating velocity increases as the angular velocity of the inner cylinder gradually increases, ranging from 31.50*mm/s* to 103.20*mm/s*.

**Table 1:** Characteristic turbulent fluctuating velocities with respect to different angular velocities of the inner cylinder.

*ω*(***rpm***)	27	42	60	90	120	150	180
$\bar{u}$(***mm/s***)	0	31.50	39.10	57.60	70.80	81.50	103.20

Similar to some reported experiments [33,45–48], this study used kaolinite (China clay) as the sediment material because of its distinct flocculation characteristics; this was done to avoid the effects of various mineral compositions in sediment on the flocculation. The grain size distribution of the primary particles of the kaolinite was analysed using a laser particle analyser (Horiba LA-920). The median size, *d*_50_, was 5.07*μm*, with a range of 0.59–23*μm*, and *d*_90_:*d*_70_:*d*_30_:*d*_10_ in the cumulative particle-size distribution was 10.27*μm*: 6.89*μm*: 3.36*μm*: 1.70*μm*. The density of the primary particle of the kaolinite sample was 2650*kg/m*^*3*^.

**Fig 1:**
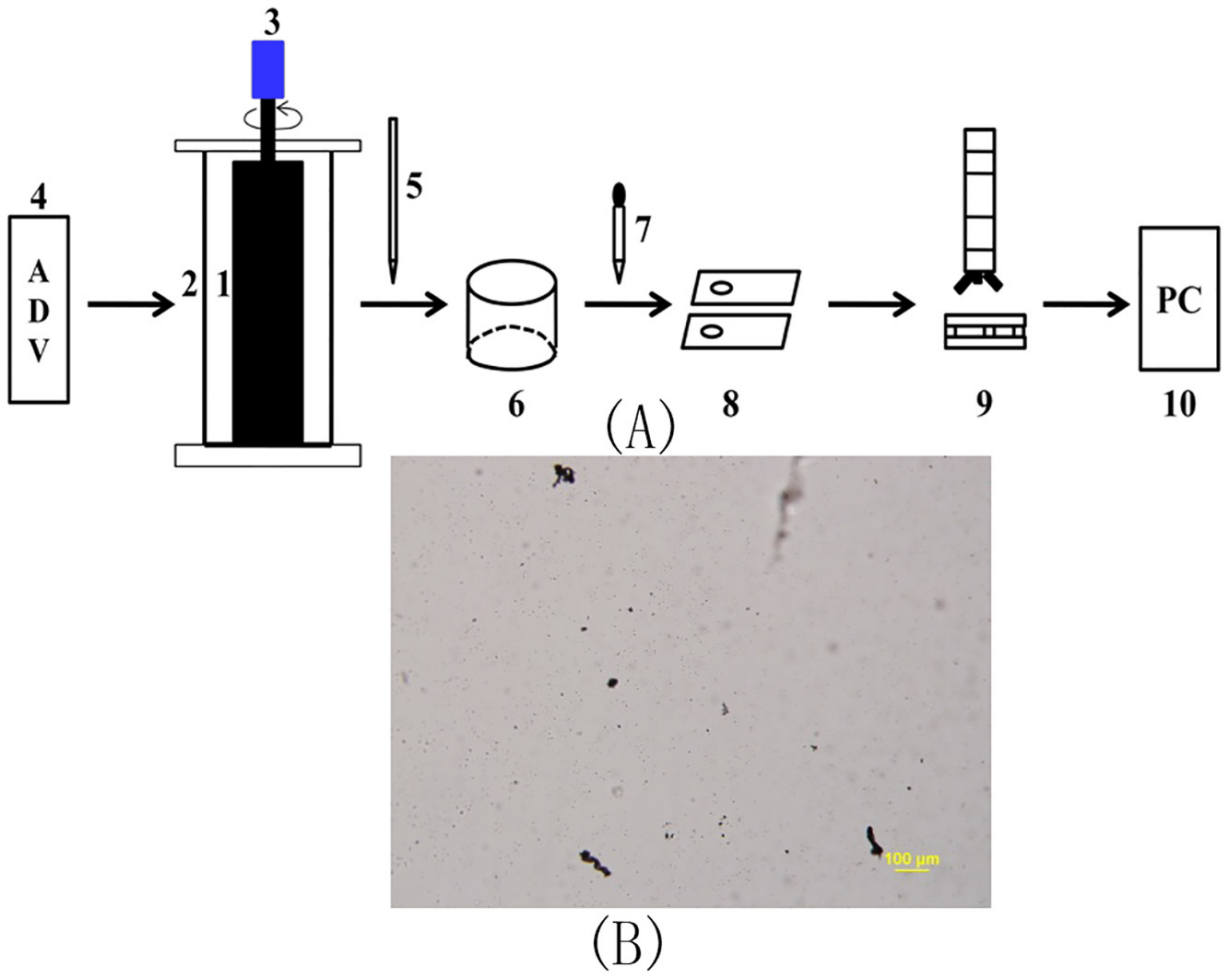
Schematic of the experimental setup (**A**) (1-the inner cylinder, 2-the outer cylinder, 3-a speed-adjusting motor with speed reduction boxes, 4-ADV, 5-a transfer pipette, 6-a volumetric flask, 7-a dropper, 8-a slide glass with a cover glass, 9-a biological fluorescence microscope with CCD, and 10-an image processing in a personal computer (PC)) and an example image (**B**) including some flocs, taken at the experimental time of 80*min* for *ω* = 90*rpm* and *ϕ* = 7.87*10^−5^ without electrolyte added to the suspension.

A schematic of the experimental setup is shown in Fig 1A. For each experiment run, we poured almost 556*L*of deionized water into the Couette-flow system and maintained the water depth at approximately 400*mm*. This water depth was determined for the convenience of a sampling operation. It was set to be smaller than the height of cylinders by 282*mm* to prevent a possible outflow of the water-sediment suspension due to an eccentric motion when the inner cylinder rotated during the experiments. After injecting an amount of kaolinite into the system with concentration by volume (it is equal to the volume of kaolinite divided by the volume of the water-sediment suspension),*ϕ* = 7.87×10^−5^, the angular velocity of the inner cylinder was adjusted to the maximum value for 5*min* to ensure that the kaolinite could be adequately suspended in the water. Moreover, to guarantee that these operations did not produce flocs, we released compressed nitrogen gas (*N*_*2*_) from the bottom of the apparatus into the system to produce a strong impulse to disrupt the possible floc production. When the angular velocity of the inner cylinder was set rapidly to the specified value using the speed-adjusting motor, the kaolinite flocculation experiment started. We extracted 1*mL*of water-sediment samples from a middle point along the vertical direction in the centre of the gap between the outer and inner cylinders using a glass transfer pipette. By gently inclining the transfer pipette, approximately 2–3 drops of sample were permitted to flow from the pipette to a small, glass volumetric flask (already filled with 1*mL*of deionized water) to dilute them by means of its self-weight. This dilution process was necessary to avoid overcrowding of flocs in each sample so that we could measure the floc size. After the dilution process was finished, we used a dropper (filled with a glass thin head end and a rubber back end) to suck an approximate 0.1*mL* diluted sample into the flask and place it carefully into the circular concave trough of a slide glass coupled with a cover glass as a final sample for a microscopic observation. A biological fluorescence microscope (connected to a high-resolution CCD) was used to take photographs of the sample. The observational system can collect images at 15 frames per second. The ratio of the pixel to physical length in the collected images was set to be 1 pixel = 1.25*μm*, and the minimum detectable particle size was simply set to be a 1×1 pixel area. Fig 1B presents an example image that includes some flocs, taken at an experimental time of 80*min* for *ω* = 90*rpm* and *ϕ* = 7.87*10^−5^ without electrolyte added to the suspension. After all images were saved on a personal computer, we calculated the projected area *A* of all flocs in a projected two-dimensional plane in each image using the software after image processing (see below). The following expression was used to estimate the equivalent spherical size (or diameter), *d*, of all flocs:$d=\sqrt{4A/\pi }$. The floc size distribution and the median floc size can be obtained based on the statistics of *d* for all flocs. The image processing included three simple steps in this study: image transform (coloured image→greyscale image→binary image), reduction of image noise using a threshold greyscale value to differentiate the flocs and the background around them (the determination of the threshold value was only based on a simple trial and error in this work), and counting of pixels encompassed by flocs(the projected area of the floc can be obtained based on this number and the ratio of the pixel to the physical length). The number of flocs in each image varied depending on the turbulent shear condition used. The floc size distribution was based on a minimum of500 flocs for each flow shear condition in this study. The experimental temperature was set at 20 ± 0.5°C.

## Results and Discussions

### 3.1. Steady state of flocculation

**Fig 2:**
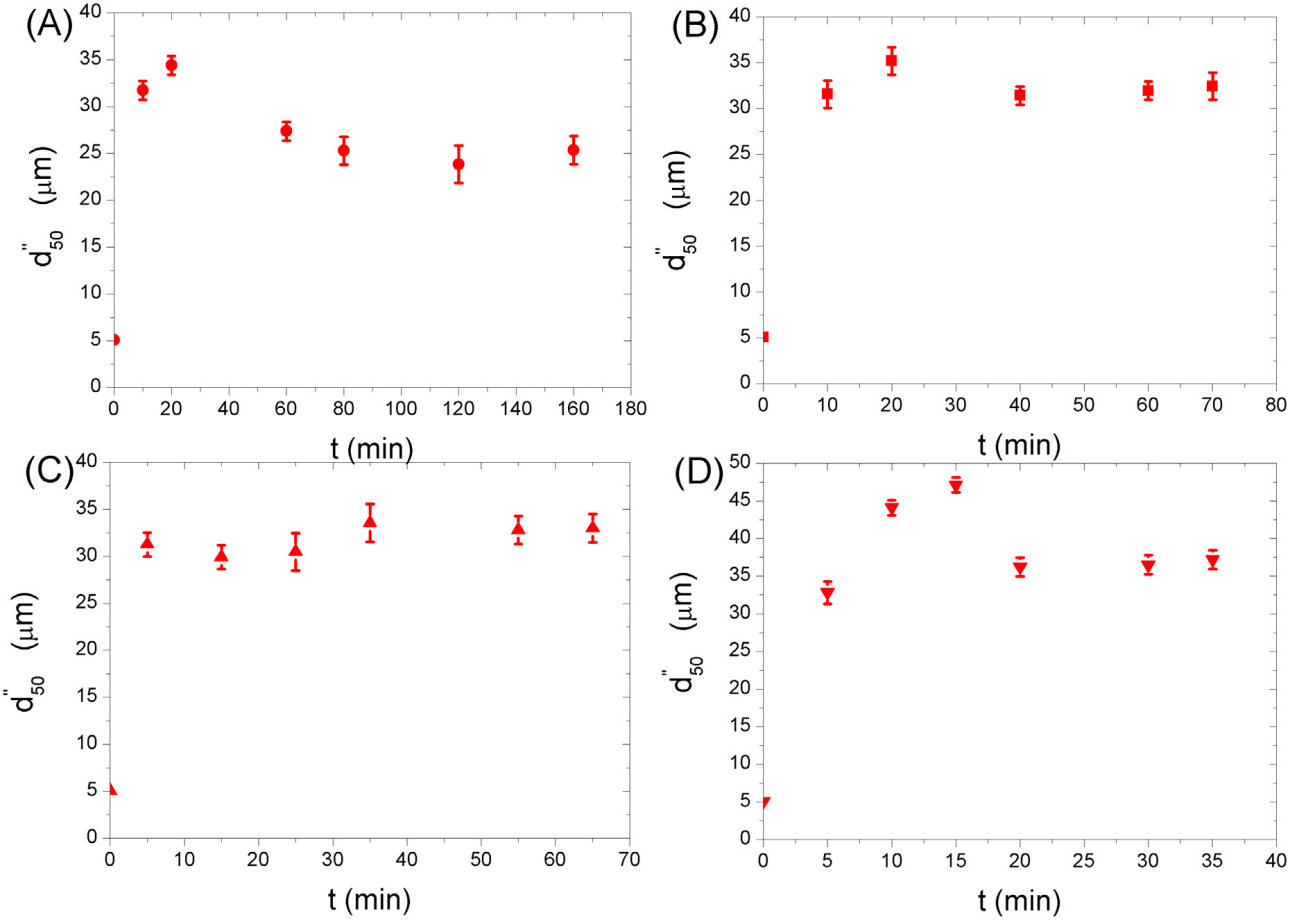
Evolution of the median floc size with the flocculation time for different characteristic turbulent fluctuating velocities (*ϕ* = 7.87×10^−5^, without electrolyte). The error bars in (**A–D**) show standard deviations of the median floc size calculated from the floc size distribution.

Fig 2A–2D show the evolution of the median floc size (or diameter),$d_{50}^{"}$,with the flocculation time, *t*,for different characteristic turbulent fluctuating velocities.

For the cases of $\bar{u}$ = 39.10*mm/s* (Fig 2A),57.60*mm/s* (Fig 2B), and 103.20*mm/s* (Fig 2D), when the flocculation experiment starts, the median floc size first increases with time up to a maximum value (0*min*<*t*<20*min*for $\bar{u}$ = 39.10*mm/s*, 0*min*<*t*<20*min*for $\bar{u}$ = 57.60*mm/s*, and 0*min*<*t*<15*min*for $\bar{u}$ = 103.20*mm/s*, respectively). This is because the flow shear increases the collision frequency between primary particles according to the Smoluchowski equation [20], consequently producing some large and porous flocs in the flow. The median floc size then decreases with time(20*min<t<*80*min*for $\bar{u}$ = 39.10*mm/s*, 20*min<t<*40*min*for $\bar{u}$ = 57.60*mm/s*, and 15*min<t<*20*min*for $\bar{u}$ = 103.20*mm/s*, respectively),which may be due to strong breakages and restructurings of those fragile flocs when they are exposed to the turbulent shear [16,30,32]. Finally, the median floc size levels off at a value after a time interval (*t*>80*min*for $\bar{u}$ = 39.10*mm/s*,*t*>40*min*for $\bar{u}$ = 57.60*mm/s*, and*t*>20*min*for $\bar{u}$ = 103.20*mm/s*, respectively), resulting from a dynamic balance between the fragmentation and the flocculation [49]. This mean floc size*-*time behaviour is the flocculation process of trend B as introduced in Section 1.

The case of $\bar{u}$ = 70.80*mm/s*is different (Fig 2C). At the beginning of the experiment, the median floc size increases with time rapidly (0*min<t<*5*min*). The main reason is that the turbulent shear between two cylinders increases the collision frequency between primary particles [20], producing some fragile, large flocs, although the breakages and restructurings of these flocs when they are subject to the flow shear may be present. When the flocculation time is longer than 5*min*, the median floc size already reaches a more or less stable level, possibly because the breakage effect (and/or the restructuring effect) caused by the flow shear balances the flocculation effect, although some fluctuations could be found in Fig 2C. For this case, no presence of the peak value of the median floc size is observed.

### 3.2. Effect of various shear conditions on steady-state floc size

#### 3.2.1. Calculation of the shear rate

To be consistent with previous experiments as introduced in Section 1, we attempted to calculate *G* based on the characteristic turbulent fluctuating velocity $\bar{u}$ in this study by using a method presented in Serra et al. (1997) [7], expressed as follows.

1. For laminar flow, *G* can be determined by using the following expression [7,21,39]:
  $$G=\frac{2\omega R_{1}R_{2}}{R_{2}^{2}-R_{1}^{2}}$$ (3)
  where *R*_1_ and *R*_2_ are the radii of the inner and outer cylinders, respectively; whereas for the turbulent flow, *G* was estimated [12,25,50–51]
  $$G=\sqrt{\frac{\varepsilon }{\nu }}$$ (4)
  where *ε* is the energy dissipation rate per unit mass (*m*^*2*^*/s*^*3*^), and *v* is the kinematic viscosity of the fluid (*m*^*2*^*/s*).
2. The exact value of the rate of energy dissipated into the system, *ε*, is difficult to determine. In the method of Serra et al. (1997), *ε* is assumed to scale as *ε*∝*u*^'3^/*ℓ*, where *u*' is a characteristic velocity fluctuation, and *ℓ* is a characteristic length [7]. It was assumed that the value of *ℓ* is scaled to the width of the gap between the two cylinders as *ℓ*∝R_*2*_ *−* R_*1*_. It seems more reasonable to use the characteristic turbulent fluctuating velocity $\bar{u}$ to replace *u*' in this study because $\bar{u}$ has been measured by ADV rather than simply using the method of Serra et al. (1997) in which *u*' was simply assumed to be a specific function of the angular velocity of the inner cylinder [7]. If the continuity between the laminar shear rate and the turbulent shear rate at the critical angular velocity, *ω*_c_ (where turbulence between the cylinders takes place, it equals 27*rpm* herein) is assumed, a proportionality ratio, *q*, can be obtained (*q* = 1.65×10^−3^ in this study).
3. Finally, *G* values corresponding to different characteristic turbulent fluctuating velocities $\bar{u}$ can be determined as long as *q* is given. *G* values corresponding to $\bar{u}$ = 31.50*mm/s*, 39.10*mm/s*, 57.60*mm/s*, 70.80*mm/s*, 81.50*mm/s*, and 103.20*mm/s* are estimated to be 9.17*s*^*-1*^, 14*s*^*-1*^, 24*s*^*-1*^, 31*s*^*-1*^, 41*s*^*-1*^, and 53*s*^*-1*^, respectively.

The above method provided an approximate estimation of the shear rate, and we used it only as a simple basis for comparing various shear effects on flocculation.

#### 3.2.2. Results

**Fig 3:**
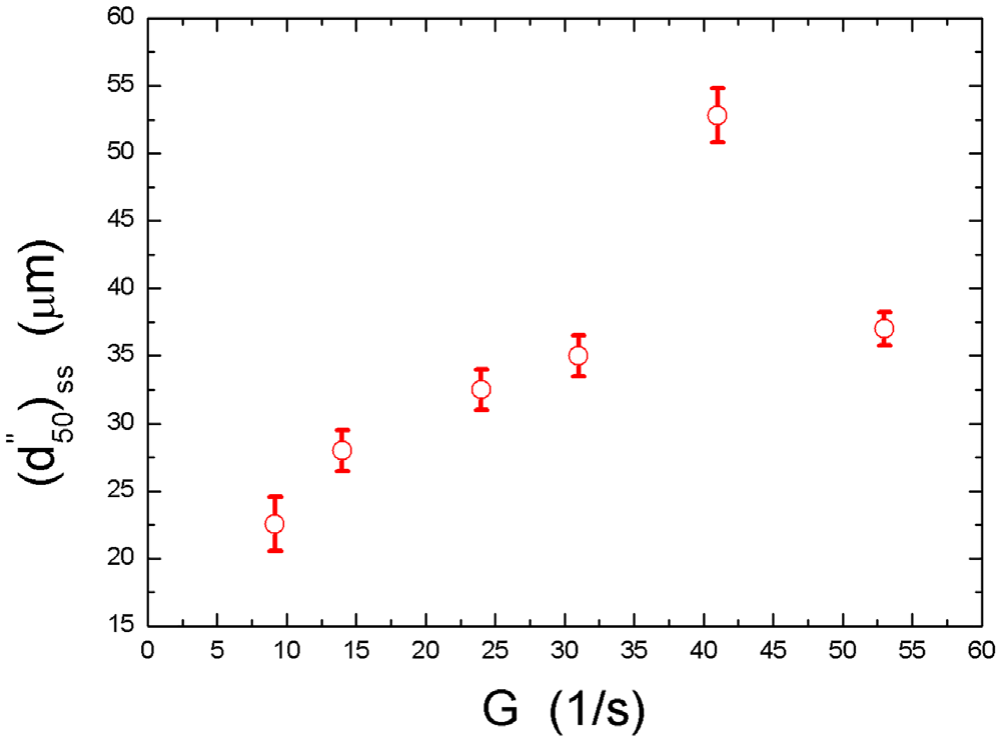
The steady-state median floc size with respect to six shear rates (*ϕ* = 7.87×10^−5^, without electrolyte). The error bars show standard deviations of the median floc size calculated from the floc size distribution.

Fig 3 shows the steady-state median floc size,${\left(d_{50}^{"}\right)}_{ss}$, with respect to six different shear rates, *G*, at *ϕ* = 7.87×10^−5^ without electrolyte added into the suspension. It can be seen that the median floc size increases with increasing *G* when *G* is less than 41*s*^*-1*^. However, it decreases with increasing *G* when *G* exceeds 41*s*^*-1*^. Based only on data in this Figure (because more trials in the range of *G* from 9.17*s*^*-1*^ to 53*s*^*-1*^ were not carried out in this experiment), the negative correlation between the median floc size and the flow shear rate only occurred at *G*≧41*s*^*-1*^. Because every steady-state median floc size is a result of the balance between the flocculation effect (induced by the flow shear) and the breakage effect (also caused by the flow shear), we can simply divide the data in Fig 3 into two different ranges as follows: a flocculation-dominated range for floc growth (9.17*s*^*-1*^<*G*<41*s*^*-1*^), and a breakage-dominated range (41*s*^*-1*^<*G*<53*s*^*-1*^).

**Fig 4:**
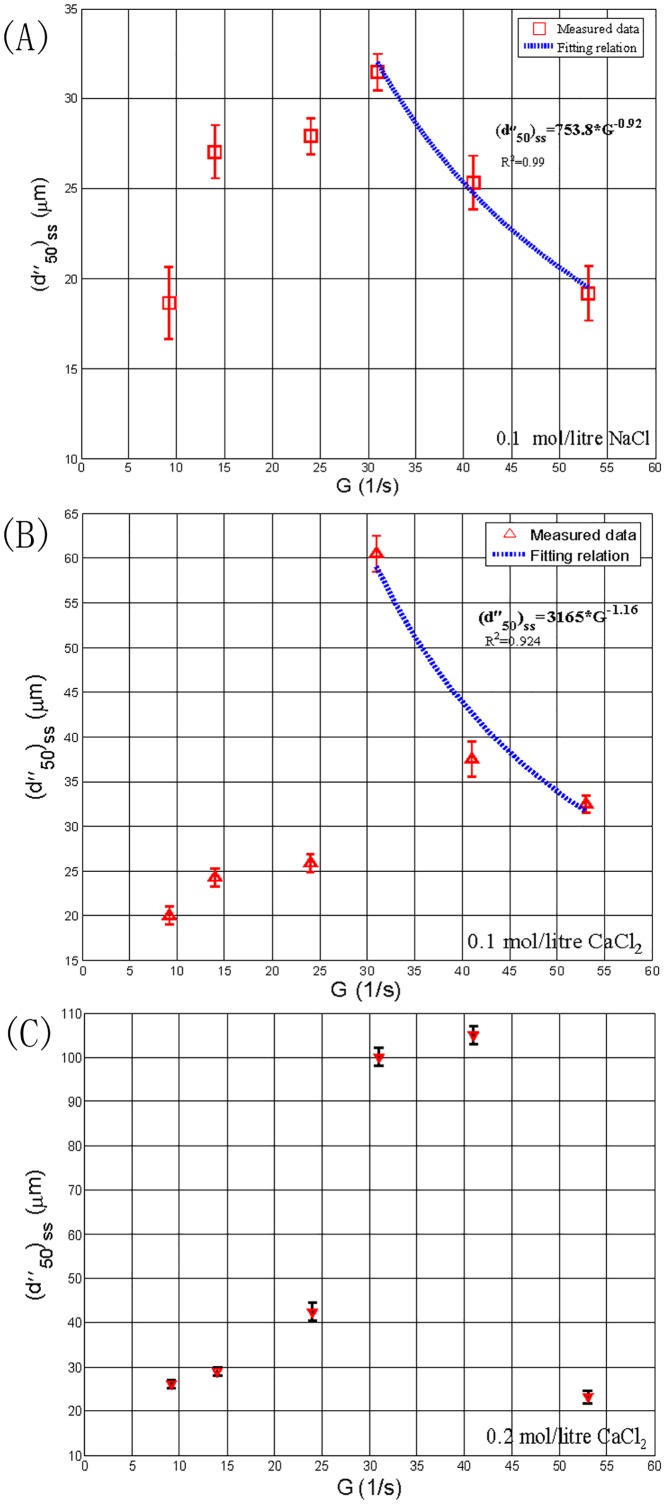
The steady-state median floc size with respect to six shear rates, with 0.1*mol/litre NaCl* (A), 0.1*mol/litre CaCl*_*2*_ (B), and 0.2*mol/litreCaCl*_*2*_ (C) (*ϕ* = 7.87×10^−5^). The error bars in (A–C) show standard deviations of the median floc size calculated from the floc size distribution.

Considering that the aforementioned discussion corresponds to the background without electrolyte added into the system, we attempted to investigate the impacts of various shear conditions on the floc size with added electrolyte. Fig 4A–4C shows the steady-state median floc size,${\left(d_{50}^{"}\right)}_{ss}$, with respect to six different shear rates, *G*, at *ϕ* = 7.87×10^-5^when 0.1*mol/litre NaCl*, 0.1*mol/litre CaCl*_*2*_, and 0.2*mol/litreCaCl*_*2*_were added into the suspension, respectively. From Fig 4A, we can find that the median floc size increases with increasing *G* when *G*≦31*s*^*-1*^, whereas a further increase in *G* appears to lead to a decrease in the median floc size. Similar to Fig 3, based only on data in this Figure, we can also divide the data into two different ranges: a flocculation-dominated range for floc growth (9.17*s*^*-1*^<*G*<31*s*^*-1*^) and a breakage-dominated range (31*s*^*-1*^<*G*<53*s*^*-1*^). Similarly, for the case with 0.1*mol/litreCaCl*_*2*_ (as shown in Fig 4B) and the case with 0.2*mol/litreCaCl*_*2*_ (as shown in Fig 4C), the flocculation-dominated range appears at the range of *G* from 9.17*s*^*-1*^ to 31*s*^*-1*^ and that from 9.17*s*^*-1*^ to 41*s*^*-1*^, respectively. The breakage-dominated range occurs at the range of *G* from 31*s*^*-1*^ to 53*s*^*-1*^ and that from 41*s*^*-1*^ to 53*s*^*-1*^, respectively.

#### 3.2.3. Comparison with reported experimental results

Some reported experimental results on steady-state median floc size with respect to different shear conditions are summarized in the paper (S2 Supporting Information). As shown in the fourth column in this table, most authors attempted to adopt a simple power function to fit the correlation between the steady-state median floc size,${\left(d_{50}^{\prime \prime }\right)}_{ss}$, and the flow shear rate, *G*, as already introduced in Section 1 using expression (1),as follows:

$${\left(d_{50}^{\prime \prime }\right)}_{ss}=cG^{-\gamma },$$ (5)

in addition to Oles (1992), in which a linear correlation was presented [21],and Zhang (1996),in which an exponential correlation was suggested [52]. However, we can find that the shear rate adopted in these experiments is large (*G*≧25*s*^*-1*^~30*s*^*-1*^), implying that it is questionable to apply expression (5) to characterize the dependence of the steady-state floc size formed in the low shear flow (especially≦25–30*s*^*-1*^) on the flow shear rate. Our experimental result (an increase in *G* tends to lead to an increase in ${\left(d_{50}^{\prime \prime }\right)}_{ss}$ at low flow shear conditions—i.e., 9.17*s*^*-1*^<*G*<31*s*^*-1*^ or 41*s*^*-1*^) shows the possibility to remedy the deficiency of these experiments.

In addition, for the breakage-dominated ranges in Figs 3 and 4A–4C, we also used expression (5) to fit them (the cases with no electrolyte and 0.2*mol/litre CaCl*_*2*_ were not shown here because there were only two measured points in their breakage-dominated ranges, and the fitting relation was not meaningful), as shown by blue dotted lines in Fig 4A and 4B respectively. The (*c*,*γ*) values for the cases with 0.1*mol/litre NaCl* and 0.1*mol/litre CaCl*_*2*_are found from Fig 4A and 4B to be (753.8, 0.92) and (3165, 1.16), respectively. As explained in Section 1,*c* is the floc strength coefficient, which is strongly dependent on the method used to measure the floc size. Thus, for a specific measurement system in this study, *c* can be used to compare the floc strength. It can be seen that improving the valence of the cation of electrolyte (*Na*^+^→*Ca*^2+^) can greatly increase the floc strength. An explanation for this behaviour will be presented in Section 3.3.

### 3.3. Effect of electrolyte condition on steady-state floc size

By comparing Fig 4A and 4B, we can find that the steady-state floc size increase as the valence of the cation of electrolyte improves (*Na*^+^→*Ca*^2+^) under some flow shear conditions, except for the cases of both *G* = 14*s*^*-1*^and 24*s*^*-1*^. By comparing Fig 4B and 4C, it can be seen that increasing the electrolyte concentration (from 0.1*mol/litre* to 0.2*mol/litre*)tends to increase the steady-state floc size under some flow shear conditions, except for the case of *G* = 53*s*^*-1*^.

A possible explanation for this behaviour is as follows. Based on the modified Smoluchowski equation [20] (see also Eqs (2) and (6) in Thomas et al. (1999)) [5], the rate of flocculation,*k*_*ij*_, between particles/flocs of sizes *i* and *j*, is proportional to the collision efficiency coefficient, *α*,and the collision frequency, *β*_*ij*_,between them and the product of their respective number concentrations, *n*_*i*_ and *n*_*j*_, as described by the following expression:

$$k_{ij}=\alpha \beta_{ij}n_{i}n_{j}.$$ (6)

For the shear-induced flocculation, the collision frequency, *β*_*ij*_, has been presented to be a specific function of the flow shear parameter in many studies (a reviewing work can be found in Thomas et al. (1999) [5]). For a given shear-induced flocculation case in this study, the parameter *β*_*ij*_ and the two parameters *n*_*i*_ and *n*_*j*_ can be simply regarded as constants. The collision efficiency coefficient, *α*, greatly depends on the binding effects of all short-range forces between two colliding particles/flocs [5], including the van der Waals attractive force and the double-layer electrostatic repulsive force. It is thus a function of the surface physico-chemical characteristics of the particle and water and the organic/inorganic compounds in the particle (no such substances in this study) [12]. According to the classical DLVO theory (Deryaguin and Landau (1941) and Verwey and Overbeek (1948), see details in Thomas et al. (1999)[5]), the total interaction force, *F*, between two colliding particles can be mainly attributed to the van der Waals attractive force, *F*_*A*_,and the double-layer electrostatic repulsive force, *F*_*R*_, by

$$F=F_{A}+F_{R}.$$ (7)

**Fig 5:**
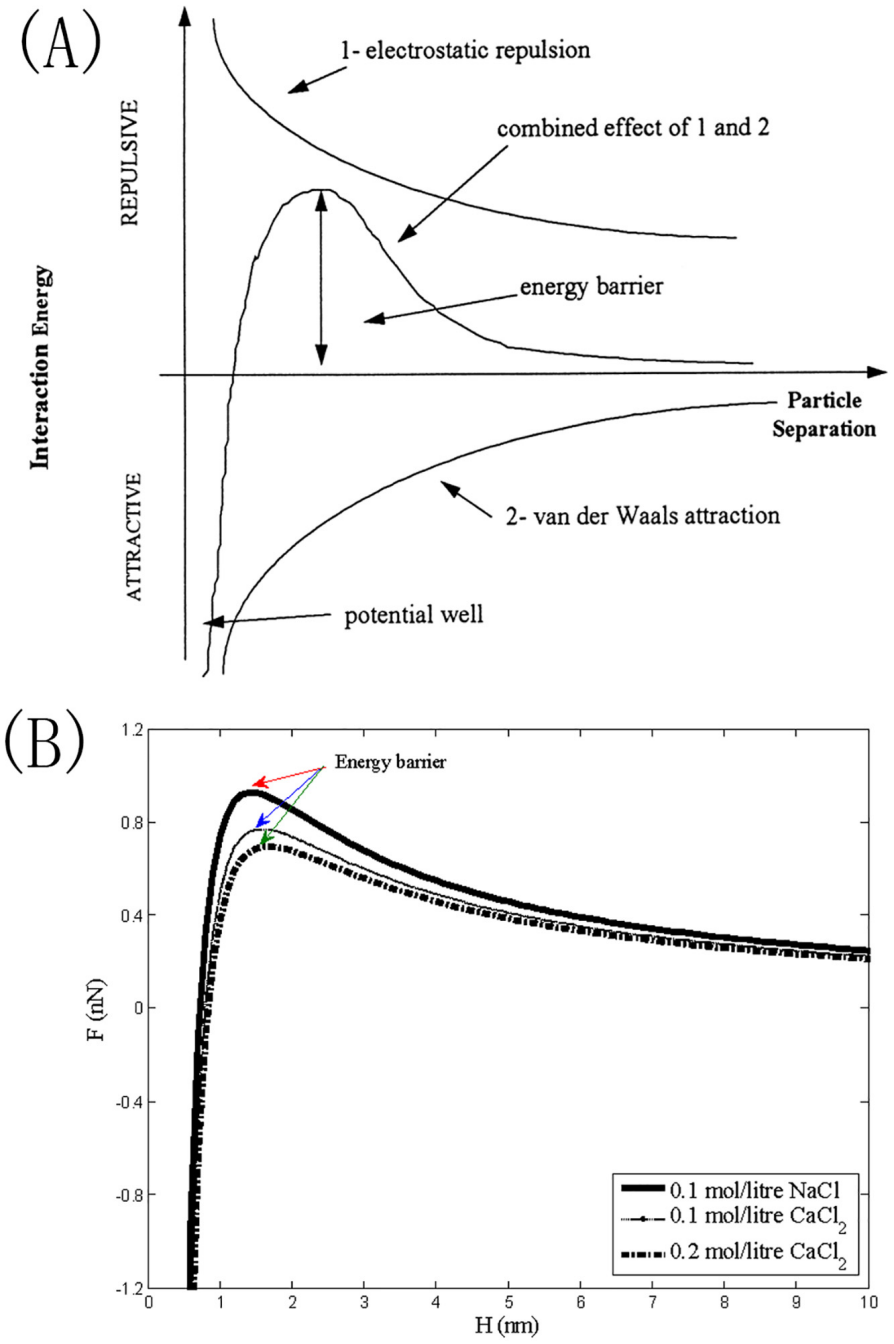
RepresentationofDLVOtheory, (A) a schematic diagram incited from Thomas et al. (1999) [5]; (B) a diagram for the cases with 0.1*mol/litre NaCl*, 0.1*mol/litreCaCl*_*2*_, and 0.2*mol/litre CaCl*_*2*_, added into the suspension.

The van der Waals attractive force, *F*_*A*_, for the case of two primary particles of the same radius can be estimated to be [53]

$$F_{A}=-\frac{A_{131}d_{50}}{48H^{2}},$$ (8)

where *A*_131_ is the effective Hamaker constant, and *H* is the separation distance between primary particles in the floc. The double-layer electrostatic repulsive force, *F*_*R*_, can be determined through the following expression for the case of two primary particles of the same radius [53]:

$$F_{R}=\frac{16\pi \varepsilon_{0}rK_{B}^{2}T^{2}}{e^{2}z^{2}H}{\left\{\frac{\text{exp}(ze\psi /2K_{B}T)-1}{\text{exp}(ze\psi /2K_{B}T)+1}\right\}}^{2}\text{exp}(-\kappa H),$$ (9)

$$\kappa ={\left(\frac{2nz^{2}e^{2}}{\varepsilon_{0}K_{B}T}\right)}^{\frac{1}{2}},$$ (10)

where *ε*_0_ is the static permittivity; *K*_*B*_ is the Boltzmann constant; *T* is the absolute temperature; *e* is the electronic charge; *z* is the valence of the cation of the electrolyte; *ψ* is the particle surface potential, which can be taken as the zeta potential; *κ*^−1^ is the thickness of the electrostatic double layer; and *n* is the electrolyte concentration. The addition of the van der Waals attractive force and the double-layer electrostatic repulsive force (Eq (8) plus Eq (9)) produce a repulsive barrier in the Figure describing the variation of the total interaction force (*F*) with respect to the particle separation distance (*H*), as schematically shown in Fig 5A.

If only the valence of the cation of the electrolyte, *z*, or electrolyte concentration, *n*, is increased, the thickness of the double layer, *κ*^−1^, will decrease (according to expression (10)). This will lead to a decreasing double-layer repulsive force *F*_*R*_ (according to expression (9)) and a descending height of the repulsive barrier (according to expressions (7) and (9) because *F*_*A*_ does not change based on expression (8)), as clearly shown in Fig 5B. Table 2 provides all parameter values for Fig 5B. Once the height of the repulsion barrier is reduced, an increasing abundance of primary particles can have the opportunity to overcome the barrier, and more adhesions between them can take place, meaning that the collision efficiency factor, *α*, is increased compared with the low-electrolyte environment. Therefore, based on expression (6), we could infer that increasing the electrolyte condition (electrolyte concentration and/or the valence of the cation) can improve the flocculation rate, being favourable to produce large flocs at the steady state of flocculation.

**Table 2:** Parameter values for presentation of the DLVO theory.

Parameter	Value	Parameter	Value
*A*_131_	1.83*10^-20^*J* (*)	*e*	1.60*10^-19^*C*
*d*_50_	5.07*μm*	*z*	1.00/2.00
*ε*_0_	6.93*10^-10^*F/m*	*ψ*	-22.37 *mV* (**)
*K*_B_	1.38*10^-23^*J/K*	*n*	0.10/0.20 *mol/litre*
*T*	293.15 *K*		

Caption:

^(^*^)^: we tused this value as presented in Zhao (2010)[54];

^(^**^)^: we used this value as measured in Yang et al. (2002) [55].

However, considering that this is the balance (between the flocculation effect and the breakage effect) that determines the steady-state floc size, it is necessary to discuss the effect resulting from the breakage (and possible restructuring) when the electrolyte condition increases. An easily understood viewpoint is that a floc will break if the shear stress exerted by the turbulent flow on the floc is larger than the bonding strength within the floc (or the strength of the floc) [4,56]. Furthermore, Tang et al. (2001) presented an expression to estimate the floc strength [53],*σ*_*T*_,as follows:

$$\sigma_{T}=1.1\frac{1-\varepsilon }{\varepsilon }\frac{F}{d_{50}^{2}},$$ (11)

where *ε* is the porosity of the floc. *F* in expression (11) is negative when the primary particles adhere, representing the fact that the attractive force *F*_*A*_ predominates over the repulsive force *F*_*R*_;in this case, the absolute value of *F* should be used [53,57]. If only the electrolyte concentration, *n*, or the valence of the cation of the electrolyte, *z*, is increased, the total interacting force *F* when primary particles adhere will be found to increase (according to expressions (7)–(10)). A quantitative calculation is as follows: the *F* values corresponding to the cases of (*z* = 1,*n* = 0.1*mol/litre*), (*z* = 2,*n* = 0.1*mol/litre*), and (*z* = 2,*n* = 0.2*mol/litre*) are estimated to be -0.58*nN*, -4.13*nN*, and -9.77*nN*,respectively, based on the parameter values in Table 2. When calculating these values, the particle separation distance (*H*) (when the adhesion between two primary particles occurs) was simply estimated to be approximately 3.1 times the thickness of the double layer (*κ*^−1^), as suggested by Yang et al. (2002) [55]. Thus, based on expression (11), we can infer that the floc strength in the high-electrolyte environment is larger, and the floc becomes more resistant to the flow shear compared with low-electrolyte environments. Furthermore, this implies that a high-electrolyte environment (electrolyte concentration, *n*, and/or the valence of the cation,*z*, are high) contributes to the production of larger flocs at the steady state of flocculation relative to a low-electrolyte environment. Attention will be paid to more electrolyte types and concentrations in future experiments, and an elaborate analysis of the combined effects of different electrolyte conditions and various turbulent shear conditions will be carried out in future research.

### 3.4. Effect of initial particle concentration on steady-state floc size

**Fig 6:**
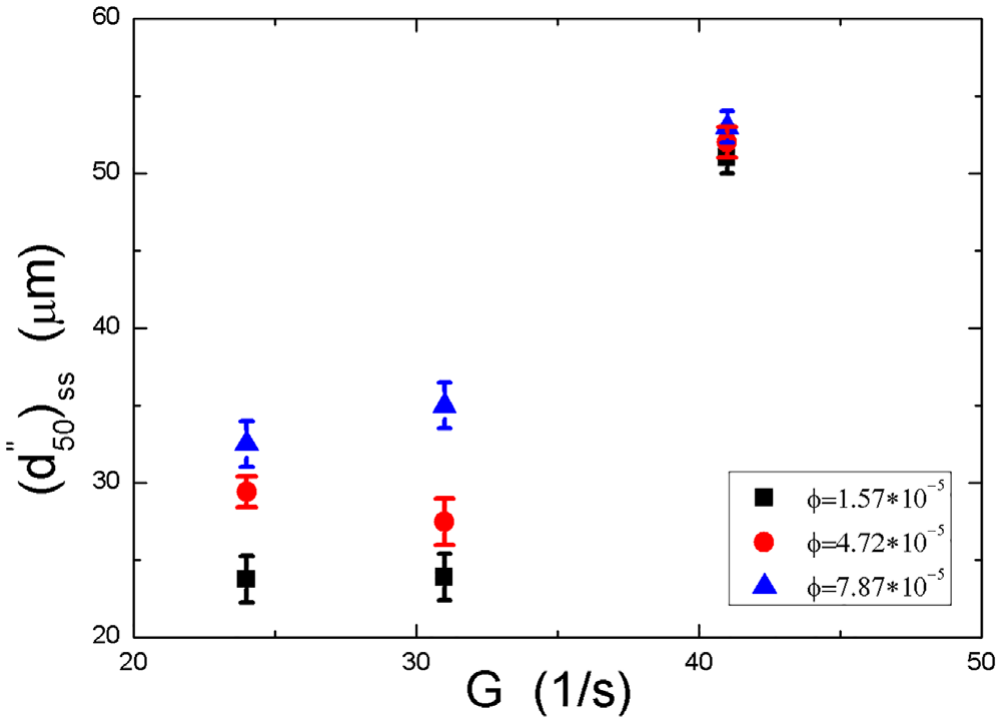
The steady-state median floc size with respect to three shear rates at different initial particle concentrations (without electrolyte). The error bars show standard deviations of the median floc size calculated from the floc size distribution.

To assess the impact of initial particle concentration on the floc size at the steady state, a change of initial particle concentration by volume, *ϕ*, from 7.87×10^−5^, 4.72×10^−5^ to 1.57×10^−5^ was carried out in this study. Fig 6 shows the steady-state median floc size with respect to three shear rates at different initial particle concentrations, without electrolyte added into the suspension. We can find that increasing the initial concentration tends to increase the steady-state floc size regardless of shear conditions. The interpretation for this behaviour is similar to that described in Section 3.3. Based on expression (6), the flocculation rate, *k*_*ij*_, between particles of sizes *i* and *j*, is proportional to the product of their respective number concentrations, *n*_*i*_, *n*_*j*_. For the present flocculating system, the collision efficiency factor, *α*, and the collision frequency, *β*_*ij*_, can be regarded as two constants because no electrolyte has been added into the suspension, and a given turbulent condition is discussed here. Moreover, as many studies have shown [2,12,14–15,17,24, 51], the breakage rate of the floc is a specific function of the flow shear. For a given shear environment, it can be simply regarded to be a constant. Thus, increasing the initial particle concentration (increasing *n*_*i*_ and *n*_*j*_) can lead to more adhesions between primary particles/small flocs and consequently produce more large flocs in the flow. More initial particle concentrations and more turbulent shear conditions will be investigated in future experiments.

## Concluding Remarks

In this study, we investigated the effect of flow shear rate on the median size of kaolinite flocs at the steady state of flocculation during a Couette-flow system. Based on data in this experiment, the results lead to the following two conclusions.

1. Increasing the flow shear rate tends to increase the steady-state floc size at low shear conditions (9.17*s*^*-1*^<*G*<31*s*^*-1*^ or 41*s*^*-1*^), showing that the flocculation effect caused by the flow shear dominates the breakage effect, which is also caused by the flow shear. However, for high shear conditions (31*s*^*-1*^ or 41*s*^*-1*^<*G*<53*s*^*-1*^), increasing the shear rate tends to decrease the floc size, showing a predominant role of the breakage effect over the flocculation effect. This experimental result includes variations of floc size with respect to shear rates, especially at low shear conditions, showing a possibility to remedy the deficiency of those reported experiments in which only the influence of the shear rate on the flocculation at a high shear condition (*G*≧25–30*s*^*-1*^) was analysed.
2. Increasing the electrolyte condition (i.e., improving the valence of the cation of electrolyte and/or increasing the electrolyte concentration) and increasing the initial particle concentration appear to lead to an increase of the steady-state floc size. Based on the DLVO theory, we propose an interpretation for these behaviours.

## Supporting Information

S1 Supporting Information All measurement data. (PDF)

S2 Supporting Information Relationship between the flow shear condition and the steady-state floc size. (PDF)
