# Supplementary material for: On the Kaolinite Floc Size at the Steady State of Flocculation in a Turbulent Flow
Source: PLoS One. 2016 Feb 22;11(2):e0148895. doi: 10.1371/journal.pone.0148895 (PMC4763281; doi:10.1371/journal.pone.0148895)
Supplement: S1 Supporting Information — (PDF) [file pone.0148895.s001.pdf]

S1 Supporting Information. All measurement data

The following is all measurement data on the radial, tangential and vertical turbulent fluctuating velocity components (u_{I1}, u_{I2}, u_{I3}) with respect to different angular velocities of the inner cylinder. The first column, ω, shows different angular velocities of the inner cylinder; the second column shows the position number where the measurement using ADV was taken; The third, fourth and fifth columns show the data, (u_{I1}, u_{I2}, u_{I3}); The last column shows the relative distances of measurement points from the bottom of the cylinders corresponding to the second column.

$\omega(rpm)$	**Position number**	u_{I2}	u_{I1}	u_{I3}	**Relative distance (above the bottom of the cylinders**
**42**	p7	5.29	7.36	5.33	0.03
	p8	2.24	2.94	2.35	0.08
	p9	2.45	3.33	2.76	0.13
	p10	2.89	3.74	3.30	0.21
	p11	2.66	3.33	2.88	0.31
	p6	2.39	3.59	2.93	0.37
	p5	2.72	4.24	3.35	0.45
	p4	2.36	3.94	3.15	0.58
	p3	2.52	3.57	2.96	0.70
	p2	2.37	3.47	2.83	0.75
	p1	2.13	3.24	2.68	0.83
**60**	p4	13.33	3.79	8.07	0.01
	p5	4.10	3.21	3.36	0.06
	p6	6.74	4.11	4.86	0.11
	p7	3.61	4.71	4.06	0.15
	p8	2.77	4.91	3.81	0.28
	p9	2.74	4.86	3.82	0.38
	p3	2.75	4.63	3.72	0.45
	p2	2.75	4.83	3.91	0.58
	p1	2.70	4.60	3.66	0.70
	p10	2.90	4.33	3.58	0.81
**90**	p4	4.46	4.57	3.96	0.01
	p6	3.83	6.39	5.13	0.12
	p7	4.90	7.05	5.94	0.15
	p8	3.79	7.18	5.54	0.28
	p9	3.79	7.20	5.53	0.38
	p3	3.94	7.02	5.59	0.45

	p2	3.99	7.05	5.76	0.58
	p1	4.04	6.95	5.60	0.70
	p10	3.89	6.47	5.24	0.81
**120**	p4	3.62	5.66	4.23	0.01
	p5	3.68	5.50	4.65	0.06
	p6	4.77	8.24	6.50	0.12
	p7	6.19	8.84	7.50	0.15
	p8	4.91	8.95	6.89	0.28
	p9	4.65	8.70	6.78	0.38
	p3	4.80	8.67	6.90	0.45
	p2	4.84	8.67	7.08	0.58
	p1	4.84	8.84	7.04	0.70
	p10	4.59	8.01	6.33	0.81
**150**	p4	4.17	7.20	5.23	0.01
	p5	4.63	7.34	6.13	0.06
	p6	5.39	10.19	8.03	0.12
	p7	6.50	10.76	8.84	0.15
	p8	5.81	10.80	8.38	0.28
	p9	5.84	10.71	8.29	0.38
	p3	5.73	10.47	8.29	0.45
	p2	5.74	10.05	8.15	0.58
	p1	5.81	10.39	8.25	0.70
	p10	5.40	9.36	7.41	0.81
**180**	p4	5.04	8.77	6.41	0.01
	p5	5.82	10.66	8.38	0.06
	p6	6.69	12.18	9.67	0.12
	p7	7.62	12.51	10.31	0.15
	p8	7.05	13.06	10.07	0.28
	p9	7.03	12.13	9.58	0.38
	p3	6.82	12.05	9.50	0.45
	p2	7.83	13.23	10.32	0.58
	p1	6.84	12.07	9.70	0.70
	p10	6.07	10.07	8.08	0.81
