# Supplementary material for: On the Kaolinite Floc Size at the Steady State of Flocculation in a Turbulent Flow
Source: PLoS One. 2016 Feb 22;11(2):e0148895. doi: 10.1371/journal.pone.0148895 (PMC4763281; doi:10.1371/journal.pone.0148895)
Supplement: S2 Supporting Information — (PDF) [file pone.0148895.s002.pdf]

S2 Supporting Information. Relationship between the flow shear condition and the steady-state floc size

The following presents the Relationship between the flow shear condition and the steady-state floc size, reported in some experiments. The first column shows the reference number; The second column shows the shear-generating apparatus; The third column shows which material was used for flocculation and which shear conditions were adopted; The fourth column shows the relationship between the flow shear rate, G , and the steady-state floc mean size, $\left(d_{50}^{''}\right)_{ss}$,reported in the literatures; The last column presents the additional information about the fitting relation between G and $\left(d_{50}^{''}\right)_{ss}$.

References	Shear-generating apparatus	Material / Shear condition	$\left(d_{50}^{''}\right)_{ss}$ (μm) and G (s^{-1})	Additional information
21	Couette-flow system	Polystyrene latex /G=25,50,75,100, 125, 150 s^{-1}	$\left(d_{50}^{''}\right)_{ss}$ = -0.27G+53.00; for ϕ =5.00*10^{-5};<br>$\left(d_{50}^{''}\right)_{ss}$ = -0.19G+32.00; for ϕ =1.00*10^{-5};	
35	Baffled stirred tank	Polystyrene particle /G=63,95,129 s^{-1}	Increasing shear increases the rate of floc breakage, resulting in a smaller average floc size at steady state	Fitting relation: $\left(d_{50}^{''}\right)_{ss}$ = 652.6$G^{-0.65}$, R^2=0.94, for alum=10.70*mg/litre*; $\left(d_{50}^{''}\right)_{ss}$ =4636$G^{-0.94}$,R^2=0.93, for alum=32.00*mg/litre*
52	Blade stirred box	Lianyungang sludge/G=40,80, 120,160,200,240 s^{-1}	$\left(d_{50}^{''}\right)_{ss}/d_{50}$ = exp(4.58-0.00155G)	
7	Couette-flow system	Latex particle /G=25,50,90,135, 195 s^{-1}	Fitting relation:<br>$\left(d_{50}^{''}\right)_{ss}$ = 378.80$G^{-0.60}$, R^2=0.91; for ϕ =1.50*10^{-5};<br>$\left(d_{50}^{''}\right)_{ss}$ = 429.70$G^{-0.63}$, R^2=0.85; for ϕ =2.50*10^{-5};<br>$\left(d_{50}^{''}\right)_{ss}$ = 872.80$G^{-0.81}$, R^2=0.81; for ϕ =5.00*10^{-5};<br>$\left(d_{50}^{''}\right)_{ss}$ = 4053.00$G^{-1.21}$, R^2=0.97; for ϕ =7.50*10^{-5};<br>$\left(d_{50}^{''}\right)_{ss}$ =2291.00$G^{-1.12}$, R^2=0.87; for ϕ =10.00*10^{-5};	
36	Induction driver rheometer	Lactose particle / τ =0~1 *Pa*	$\left(d_{50}^{''}\right)_{ss}$ = 0.43 (E_b/τ)$^{1/3}$, where E_b is the breaking energy, and τ is the flow shear stress.	

37	Ring-driven annular flume	Weir Quay mud /G=24.2,30.7,37.8, 45.2 s^{-1}	$\left(d''_{50}\right)_{ss}$ = 6.72(ϕG)$^{-0.45}$	
25	Baffled batch vessel	Activated sludge /G=19.4,58.2,113,182,261,346,444 s^{-1}	Fitting relation:<br>$\left(d''_{50}\right)_{ss}$ = 388.60$G^{-0.43}$, R^2=0.97	
58	Jar-test vessel	Bentonite particle/G=35,60,90,180, 360 s^{-1}	Maximum floc size $\propto G^{-1}$ for laminar flow; Maximum floc size $\propto G^{-2/3}$ for turbulent flow;	
33	Impeller reactor	Kaolin clay particle/G=40,50,70,90 s^{-1}	As G increased, the mean size decreased	
59	Impeller tank	Bentonite particle/G=30,50,75,100,150,200,250,300 s^{-1}	Fitting relation:<br>$\left(d''_{50}\right)_{ss}$ = $10^{3.01}G^{-0.50}$;<br>$\left(d''_{50}\right)$ = $10^{2.49}G^{-0.30}$;	
60	Impeller jar	Kaolin/G=29.1,67.7,99.7,172.1,253.4,342 s^{-1}	Fitting relation:<br>$\left(d''_{50}\right)_{ss}$ = $10^{3.37}G^{-0.66}$;<br>$\left(d''_{50}\right)$ = $10^{2.24}G^{-0.16}$;	
61	Couette-type reactor	The reservoir water/G=40,60,80,100,150,200,250,300,350 s^{-1}	Fitting relation:<br>$\left(d''_{50}\right)_{ss}$ = $10^{5.47}G^{-1.44}$;<br>$\left(d''_{50}\right)$ = $10^{2.60}G^{-0.28}$;	
30	Taylor-Couette reactor	Impurity in raw water and ferric hydroxide/G=21.2,38.7,58.9,79.8,102.3,149.1,202.2,252.8,300.5,347.9 s^{-1}	Fitting relation:<br>$\left(d''_{50}\right)_{ss}$ = $10^{4.66}G^{-1.12}$;<br>$\left(d''_{50}\right)_{ss}$ = $10^{2.46}G^{-0.28}$;	

References

58. Bouyer D, Line A, Cockx A, Do-Quang Z. Experimental analysis of floc size distribution and hydrodynamics in a jar-test. Chemical Engineering Research and Design. 2001;79(8):1017-24.
59. Bouyer D, Line A, Quang ZD. Experimental analysis of floc size distribution under different hydrodynamics in a mixing tank, AIChE journal.2004; 50(9):2064-81.
60. Li T, Zhu Z, Wang D, Yao C, Tang H. Characterization of floc size, strength and structure under various coagulation mechanisms. Powder Technology, 2006;168, 104-10.
61. Mutl S, Polasek P, Pivokonsky M, Kloucek O. The influence of G and T on the course of aggregation in treatment of medium polluted surface water. Water Science and Technology. 2006; 6(1):39-48.

S2 Supporting Information. Relationship between the flow shear condition and the steady-state floc size
